# Gaining a comprehensive understanding of antimicrobial resistance in dental education: a mixed methods study protocol

**DOI:** 10.3389/fpubh.2026.1943489

**Published:** 2026-09-14

**Authors:** Belinda Nicolau, Isaac Montgomery, Quan Nha Hong, Alissa Levine, Christophe Bedos, Sreenath Madathil, Robert Durand, Aimée Dawson, Fatiha Chandad, Wendy Somerville, Annie Mao, Safae Benali Lietefti, Fernanda Cristina Petersen, Roger Junges, Mary Ellen Macdonald

**Affiliations:** 1 Faculty of Dental Medicine and Oral Health Sciences, McGill University, Montréal, QC, Canada; 2 Department of Primary Care and Rural Medicine, Texas A&M University, Bryan, TX, United States; 3 School of Rehabilitation, Université de Montréal, Montréal, QC, Canada; 4 Faculty of Dental Medicine, Université de Montréal, Montréal, QC, Canada; 5 Faculty of Dentistry, Université Laval, Québec, QC, Canada; 6 Faculty of Dentistry, University of Oslo, Oslo, Norway

**Keywords:** antibiotic prescriptions, antimicrobial resistance, antimicrobial stewardship, dental education, dental practice, dental school, health policy

## Abstract

**Background:**

Antimicrobial resistance (AMR) is one of the most pressing global health challenges of the 21st century, driven in part by suboptimal prescription of antibiotics. Most antibiotic use worldwide occurs in outpatient settings, including dental practice, where dentists account for approximately 5–11% of all prescriptions. Educational environments play a critical role in shaping prescribing behaviors early in professional development, and interventions implemented during undergraduate training are more effective at changing clinical practice than those introduced later in clinical careers. However, research on how dental curricula, institutional culture, and stakeholder perspectives influence antimicrobial stewardship education is limited. Therefore, this study proposes a comprehensive evaluation of antibiotic prescribing practices and AMR education across all dental schools in Québec, Canada.

**Methods:**

Using a convergent mixed methods design, the project integrates quantitative and qualitative approaches to examine knowledge, attitudes, prescribing patterns, and educational content about antibiotics and AMR. Quantitative components include a cross-sectional survey of dental students and a retrospective analysis of antibiotic prescriptions within undergraduate clinics. Qualitative components include focus groups with students and interviews with clinical instructors and academic administrators, guided by the Health Belief Model and the Awareness, Desire, Knowledge, Ability, and Reinforcement (ADKAR) model. A curricular document analysis will further explore how antimicrobial stewardship principles are taught and implemented across institutions. Findings will be integrated through a mixed methods matrix to identify convergences, gaps, and opportunities for educational change.

**Discussion:**

This work advances current research by moving beyond traditional survey-based assessments toward a holistic, multi-stakeholder understanding of antibiotic prescribing in dental education. By linking student and educator insights with clinical practice data, the study aims to generate actionable knowledge to aid curriculum developers, clinical practitioners, and policy shapers to optimize antibiotic use and mitigate antimicrobial resistance.

**Study protocol registration:**

https://cihr-irsc.gc.ca/e/193.html, # PJT183763.

## 1 Introduction

Antimicrobial resistance (AMR) is a growing public health emergency that has been identified by the World Health Organization (WHO) as one of the top ten threats to global health (1, 2). If no action is taken, by 2050 an estimated 10 million deaths could occur annually due to antimicrobial-resistant pathogens (3) and up to $100 trillion could be lost to increased healthcare needs and reduced economic productivity (3). As pathogens become increasingly resistant, modern medical procedures, including caesarean sections, organ transplantation, immunosuppressive chemotherapy, and pacemaker implantation surgery, become riskier due to their dependence on effective antibiotics (4). The factors contributing to the steep rise of AMR are multifarious (5), spanning systems in human, animal, and environmental health (6). Accordingly, the WHO has called for a sustained One Health response to the AMR problem (7). Within human medicine, a major contributor to AMR is the overprescription of antibiotics (3). Most antibiotic consumption occurs in outpatient primary care settings, including dental practice (8, 9). Dentists are among the leading prescribers, accounting for over 10% of antibiotic use in North America (10–12).

Dentists, then, are key players in the One Health strategy to address AMR. However, efforts to reduce antibiotic prescribing have yet to substantially change dental practice. For example, in British Columbia, Canada, despite updated clinical guidelines that narrowed indications for prophylactic antibiotic use, dental antibiotic prescribing increased by 62.2% between 1996 and 2013, rising from 6.7 to 11.3% of all prescriptions, while there was a 12.7% reduction in the overall rate of prescribing antibiotics across all health disciplines during the same period (12). Furthermore, recent insurance claims data suggest that more than 80% of dental antibiotic prescriptions may be unnecessary (13).

Surveys among dental practitioners have highlighted multiple antibiotic prescription behaviors requiring improvement (14–17). Among the most significant contributors to inappropriate antibiotic use is insufficient knowledge and a tendency among dentists to prescribe when faced with uncertainty (18). Antibiotic prescribing decisions are strongly influenced by early professional education and workplace culture (19–21). Since formative educational interventions have been shown to be more effective than attempts to modify entrenched prescribing habits later in clinicians’ careers (22), strategies aimed at improving antimicrobial stewardship should focus on early educational stages when professional behaviors are still forming (22, 23). This makes continuous development of dental curricula essential in response to evolving global health challenges.

Within dentistry, there is limited information regarding students’ and clinical instructors’ perceptions of AMR, as well as the role of academic leadership and curricular content in antimicrobial stewardship education. Collectively, these findings underscore the need to examine prescribing behaviors throughout dental education (13–15, 23). These issues require a holistic approach that considers classroom teaching, clinical training, and professional culture (21).

To explore these complexities, we propose a comprehensive study of AMR and antibiotic prescribing across the three dental schools in Québec, Canada. This research aims to inform curriculum development, support antimicrobial stewardship, and contribute to sustainable antibiotic use in dentistry, with implications for broader public health policy and practice. This study draws on two complementary theoretical models: the Health Belief Model, used to examine students’ perceptions, attitudes, and knowledge (24, 25), and the Awareness, Desire, Knowledge, Ability, and Reinforcement (ADKAR) model, used to explore perspectives of professors, clinical instructors, and academic administrators regarding educational change and optimal prescribing practices (26, 27). The ADKAR model focuses on inspiring change in large organizations at the individual level, not imposing change (28, 29), making it a helpful sensitizing construct for this work.

This project originated from a collaboration with the University of Oslo, during which a validated survey instrument was administered across several international dental schools, including McGill University (Québec, Canada) (30, 31). Findings suggested variability in AMR awareness and confidence in antibiotic prescribing among students, highlighting the need for a comprehensive mixed methods approach (31).

### 1.1 Study objectives

We therefore propose the following five objectives:

#### 1.1.1 Quantitative objectives

(i) To evaluate dental students’ perceptions, knowledge, and attitudes regarding antibiotic prescribing and AMR; (ii) To assess antibiotic prescribing practices within undergraduate dental clinics through retrospective analysis of dental records.

#### 1.1.2 Qualitative objectives

(iii) To explore students’ perceptions, attitudes, beliefs, and recommendations related to antibiotic prescribing and AMR; (iv) To identify facilitators, barriers, and opportunities for change from the perspectives of clinical instructors and academic administrators; (v) To examine how antibiotic prescribing practices and AMR are taught within dental education curricula across the participating dental schools.

## 2 Methods and analysis

### 2.1 Study design

This project will employ a convergent mixed methods design (32). Mixed methods address both “what?” and “how?” questions and elucidate complexity within health experiences and behaviors (33). In this design, the quantitative and qualitative strands are carried out during the same phase of the study: data are collected and analyzed concurrently but independently, and the two sets of findings are merged at the interpretation stage.

#### 2.1.1 Quantitative component

##### 2.1.1.1 Aim (i): cross-sectional survey of dental students

A cross-sectional web-based survey will be conducted to evaluate students’ perceptions, knowledge, and attitudes regarding antibiotic prescribing and AMR.

*Recruitment and sample size*: All dental students enrolled at the three dental schools in Québec, Canada, will be invited to participate in the anonymous web survey using the Nettskjema platform^1^, which is hosted on secure servers in Norway. Recruitment procedures will include emails and invitations during scheduled class sessions. As this is a census-style survey, all enrolled students will be invited to participate; therefore, no formal power calculation is required.

*Questionnaire:* The questionnaire consists of fourteen closed-ended, multiple-choice questions divided into three sections: (i) Perceptions and knowledge (6 questions); (ii) Experiences and confidence (4 questions); and (iii) Improvement and continuing education (4 questions), plus an open-ended fifteenth question for participants to express their opinions about the questionnaire topics. The instrument was originally developed in English by a multidisciplinary group of research and education professionals in microbiology and AMR and allows for assessment standardization and comparative studies across multiple regions and student cohorts over time. Following content and face validation (31), the instrument was then translated to multiple other languages by native speakers and back-translated into English by independent experts. The back-translated versions were then compared to the original for accuracy and minor adjustments. The final version was pre-tested with a convenience sample of participants with native fluency in each of the languages represented (30, 31). For this study protocol, translation of the instrument to French will similarly be performed to administer it across all dental schools in Québec, Canada.

*Data analysis:* The data will be mapped using descriptive statistics. Categorical variables will be summarized as frequencies and proportions, ordinal items as medians and interquartile ranges, and continuous variables as means and standard deviations. Results will be presented overall and stratified by institution and year of study. Partially completed questionnaires will be retained in the analysis: for each item, the number of valid responses will be reported and percentages calculated using the available responses as the denominator, and the extent and pattern of item-level missing data will be described. Graphical analyses will be generated to illustrate the results. Analyses will be conducted using RStudio.

##### 2.1.1.2 Aim (ii): retrospective assessment of prescription practices

A retrospective review of antibiotic prescriptions over the last 5 years from undergraduate dental clinics will be conducted using electronic clinical records from the three participating schools.

*Data collection:* A standardized database will be created using FileMaker software to allow for structured data entry. Variables collected will include patient age, sex, procedure type, antibiotic prescription details, prescriber information, duration of therapy, and infection outcomes at 14 and 90 days post-procedure. Research assistants will be trained to ensure consistent data extraction and entry procedures. Quality control measures will include duplicate entry of 10% of the dataset to assess agreement.

*Data analysis:* The principal outcomes will be the frequency and characteristics of antibiotic prescriptions, including antimicrobial agent and, where available, indication, dose, frequency, and duration. Analyses will be descriptive and conducted separately for each institution to inform the development of context-specific interventions; formal comparisons among institutions and classification of prescriptions as appropriate or inappropriate are not planned.

The overall prescribing rate will be reported as the number of antibiotic prescriptions per 100 visits, with 95% confidence intervals, together with the distribution of agents, doses and durations. Results will be summarized overall and by institution and presented graphically. The completeness of each extracted variable will be quantified and reported. Records with missing fields will be retained: missing values will be reported as a distinct “not recorded” category, as incomplete documentation is itself relevant to antimicrobial stewardship and to the design of the intervention. Analyses will be conducted using RStudio.

#### 2.1.2 Qualitative component

##### 2.1.2.1 Aim (iii): student perspectives

A qualitative descriptive methodology will be employed using focus group interviews (34, 35).

*Recruitment:* Using a combination of recruitment strategies, including classroom announcements, peer-to-peer dissemination through student representatives, and support from faculty collaborators, we will recruit students at all three dental schools in Québec. A sampling matrix will ensure maximum variation based on gender, age, and year of study.

*Sample size:* Focus groups will include six to eight participants, which is the number of people typically recommended for effective group discussion (36). One focus group will be conducted for each year of the dental program, for a total of thirteen focus groups across three schools. Sessions will be moderated by an experienced facilitator and supported by a co-moderator who will record field notes. Discussions will be audio-recorded and transcribed verbatim.

*Data collection:* Focus groups will be conducted using interview guides developed following the Health Belief Model. This model (24, 25) aims to predict health behavior through underlying constructs such as risk and self-efficacy. To investigate actual and intended health behavior practices, the model requires minor adaptations, such as assessing awareness of risk rather than risk directly. Our adaptation of the Health Belief Model is outlined in Table 1. The interview guides will structure the exploration of dental students’ perceptions of the risks and benefits of addressing AMR as well as their confidence in applying what they have learned in the classroom and student clinic to engage in optimal antibiotic-prescribing practices. Students will also be asked about the health needs and beliefs of their patients. Finally, recommendations regarding curricular changes will be gathered, either from a revealed lack of knowledge and confidence that implies a need for change or from explicitly proposed modifications to the curriculum.

**Table 1:** Operational definitions of the Health Belief Model.

Construct	Operational definition
Risk susceptibility	Perceived importance of antimicrobial resistance (patient and societal levels)
Risk severity	Perceived danger of failing to prescribe or over-prescribing (patient and societal levels)
Self-efficacy	Confidence in knowing when and how to prescribe antibiotics, ability to communicate with patients to ensure patient well-being, stewardship
Benefits to action	Perceived facilitators (patient and societal levels)
Barriers to action	Perceived obstacles, including patient demands
Cues to action	Direction from clinical instructors, proposed changes to clinical or classroom teaching

*Data analysis:* Data will be analyzed using thematic analysis (37), combining (a) analysis through deductive coding drawing upon the Health Belief Model (24, 25) interview domains and (b) analysis through inductive coding generated from novel or unanticipated findings. To enhance analytic rigor and consistency, members of the research team will independently review an initial subset of transcripts and collaboratively develop and refine the coding framework. Coding definitions and decisions will be documented, and discrepancies in interpretation will be discussed during regular analytical meetings. An audit trail, including changes to the coding framework and analytic memos, will be maintained throughout the study. Comparable procedures will be applied to the analysis of student focus groups and interviews with instructors and administrators. Final analysis is expected to offer a refined understanding of each central issue.

##### 2.1.2.2 Aim (iv): academic and clinical instructor perspectives

Semi-structured individual interviews will be conducted with clinical instructors and academic administrators.

*Recruitment:* Participants across all three universities will be recruited via email and in-person contact. Maximum variation sampling will consider gender, age, and years of clinical experience to ensure participation is reflective of the varied instructor backgrounds. We will purposively sample instructional faculty who teach about antibiotics in the classroom or clinic. For academic administrators, the focus will be on those involved in developing or overseeing the curriculum, including deans, associate academic undergraduate deans, associate clinical deans, and division heads.

*Sample size:* Approximately 17–18 clinical instructors and academic administrators across institutions will be interviewed. A systematic review of empirical studies that assess saturation in qualitative research found that most studies reached saturation between 9 and 17 interviews (38). Data collection and analysis will proceed iteratively, and recruitment may be extended if the planned sample does not provide sufficient information power or thematic depth to address the study objectives.

*Data collection:* Semi-structured in-depth interviews exploring opportunities and challenges in AMR education will be conducted and audio-recorded. The recordings will be transcribed verbatim for subsequent analysis. Interview guides will include questions related to the underlying possibilities for change as defined in the ADKAR model (26), which has increasingly been employed in studies of health management (27). The five constructs within the ADKAR model will be adapted to fit not only these participants’ understanding of AMR but also their assessment of what other health professionals or future health professionals know and do. Definitions of each component of the model that will guide the interviews of clinical instructors and academic administrators are detailed in Table 2.

**Table 2:** Operational definitions of the ADKAR model.

ADKAR construct	Operational definition for interviews with clinical instructors	Operational definition for interviews with administrators
A: Awareness	Experience at the undergraduate clinic and private practice	Awareness of undergraduate classroom and clinical experience
D: Desire	Willingness to effect change through teaching	Willingness to direct change through curriculum review
K: Knowledge	Knowledge of students; current teaching	Curriculum content
A: Ability	Efficacy in teaching	Perceived efficacy of teaching
R: Reinforcement	Application in clinical practice	Observed application in clinical practice; continuing education

*Data analysis:* Analysis of transcribed interviews will follow a hybrid thematic analysis: (a) deductive coding drawing upon the ADKAR domains and (b) inductive coding generated from novel or unanticipated findings. At least three researchers will be involved in the analysis, with regular discussion among the team members. Final analysis is expected to offer a better understanding of each central issue. Data from interviews with instructors and administrators will be compared with student focus group data.

##### 2.1.2.3 Aim (v): curricular document analysis

Curricula mapping will examine the distribution and coherence of AMR content across courses, including exam materials and teaching plans. Teaching materials related to microbiology, AMR, and clinical practice will be reviewed using document analysis methods (39).

*Data collection:* Learning outcomes and outlines of each dental course will be collected and reviewed across each program, from basic sciences to clinical training. Courses referencing AMR, antimicrobial stewardship, or related competencies will undergo slide-by-slide analysis using course slides obtained with approval from the relevant curriculum department.

*Data Analysis:* Curriculum content will be mapped using the WHO Competency Framework for Health Worker’s Education and Training on AMR (40). Learning objectives will be extracted from the slides and mapped to the relevant AMR competencies, documenting when and in which educational context competencies are taught and how they are positioned across training stages. For each objective, we will assign a predefined coverage level using the WHO AMR tool categories (well covered, minor improvement needed, major improvement needed, not covered). Extracted learning objectives will be independently coded by two trained reviewers using predefined criteria aligned with the WHO competency framework. Disagreements will be resolved through discussion, and the coding manual refined iteratively to ensure consistent interpretation across institutions. Focus groups and individual interview data will be linked to the curricular material. For example, if students recall learning about optimal prescription practices in a pre-clinical session during a focus group, their presentation in the course outline will be examined.

#### 2.1.3 Mixed analysis

Mixed methodology is distinguished from other methodologies by the points of interface between qualitative and quantitative research, emphasizing each component’s unique value (41). In this project, mixed methods integration will occur at the level of interpretation and reporting, where the results of each data set (qualitative and quantitative) will be compared.

Following Creswell (32), separately analyzed data will be combined in a joint display matrix to compare the results of the five objectives. The emergent qualitative and quantitative findings will be used to fill out the matrix, where possible, analyzing convergence, complementarity, uniqueness, and discrepancy (41). Discordant findings will be explored, rather than resolved by privileging one data source, and will be interpreted in relation to institutional context, participant perspectives, and differences in the phenomena captured by each method.

Rather than viewing each component as an independent outcome, integrated analysis will enable interpretation of how students’ knowledge and attitudes relate to observed prescribing practices, educational content, and the perspectives of educators and academic leaders. This comprehensive approach is expected to provide a richer understanding of the complex factors influencing antimicrobial stewardship in dental education than any single method could achieve.

## 3 Discussion

A central premise of this study is that antibiotic prescribing behaviors are not solely driven by clinical knowledge but are deeply embedded within educational experiences, professional identity formation, and institutional culture. Previous literature suggests that inappropriate prescribing may originate during undergraduate training, when clinical reasoning patterns and professional norms are first established (18, 42, 43). Prescribing decisions are influenced not only by knowledge but also by perceptions of risk, professional expectations, and experiences within training environments, where uncertainty and perceived pressure may contribute to overprescribing (10, 18, 19, 25).

The Health Belief Model provides a theoretical framework for exploring how students’ perceptions of AMR, confidence in prescribing, and self-efficacy influence their prescribing intentions (25). This approach enables examination of how awareness of AMR risk and potential benefits of stewardship translate into clinical decision-making. Simultaneously, the incorporation of the ADKAR framework offers insight into institutional readiness for change, allowing exploration of the barriers, opportunities, and reinforcement strategies related to curricular reform perceived by educators and administrators (26, 27). Integrating these behavioral and organizational models is a key strength of this protocol, as it bridges individual cognitive processes with systemic change dynamics. By acknowledging clinicians’ dual responsibility to provide optimal care for individual patients and preserve antibiotic efficacy at a societal level, the study aims to identify leverage points for strengthening stewardship during professional education.

This study will examine the relationship between students’ theoretical understanding of AMR and their confidence in applying stewardship principles in clinical settings. Previous investigations suggest that such discrepancies may exist (18, 19), but whether they are observed in this context remains to be determined.

Best practices in health education research require triangulation of findings across multiple data sources to optimally understand behavioral and organizational processes (41). Existing literature validates each approach proposed in this study: student surveys (30, 31, 44–46), student focus groups (47, 48), faculty and practitioner interviews (49–51), retrospective dental antibiotic prescription analysis (9), and mapping of dental antibiotic teaching (52). In contrast to these single-method, single-stakeholder studies, our protocol builds upon and significantly advances this literature through its integrated mixed methods approach, which yields a whole that is greater than the sum of its parts (53).

Importantly, this study extends beyond student perspectives by incorporating the voices of clinical instructors and academic leadership, as well as investigating clinical prescription practices and educational materials. Educational culture, role modelling, and clinical supervision have been identified as important influences on prescribing habits (19, 21, 25). Examining curricular materials and real-world practice alongside stakeholder interviews may provide a holistic understanding of how classroom teaching, clinical learning environments, and institutional priorities interact to shape antimicrobial stewardship practices.

Understanding the interacting factors that shape these formal and informal learning environments and institutional practice has vast potential to inform policy and curriculum development at institutional and national levels. Dentistry has historically been underrepresented in antimicrobial stewardship research compared with other healthcare disciplines, despite its substantial contribution to outpatient antibiotic use (9, 10). By generating evidence that integrates educational, behavioral, and clinical perspectives, this study may help position dental education as a key driver of stewardship innovation.

Ultimately, optimizing antibiotic prescribing in dentistry requires more than knowledge dissemination; it demands transformation of clinical culture, institutional priorities, and educational strategies. By integrating behavioral science, organizational change models, and clinical data, the proposed research represents an important step toward developing sustainable antimicrobial stewardship practices within dental education and generating the evidence needed to inform future implementation and evaluation of educational interventions.

### 3.1 Limitations

Inherent in the kind of participant-based study proposed are several limitations. Participation in surveys and qualitative interviews will be influenced by self-selection bias, potentially overrepresenting individuals with background knowledge of AMR. Variability in clinical documentation practices across institutions may affect data consistency in retrospective analyses: prescriptions cluster by prescribers and patients, and the reported confidence intervals will be unadjusted for this clustering. They are therefore presented as a descriptive indication of precision only. Any future comparative analysis would require methods accounting for the clustering of these data. Furthermore, findings will reflect the specific regulatory and educational context of Québec dental schools, which could affect generalizability to other settings. Nevertheless, the detailed methodological framework and theoretical underpinnings of this study enable its interpretation within or adaptation to diverse educational environments.

## 4 Conclusion

A One Health approach is needed to reverse the concerning global trend of AMR development. Dentists are key players in this approach due to their significant role in antibiotic prescribing. Since data show that undergraduate dental education and early professional development largely shape antibiotic prescribing practices, understanding the influence of these systems is indispensable. This study advances the current understanding of antimicrobial stewardship in dentistry by adopting a comprehensive mixed methods approach that integrates stakeholder perspectives, educational analysis, and clinical data. The results of this project will inform strategies that promote optimal and sustainable antibiotic use in dentistry.

## Footnotes

1
https://nettskjema.no/?lang=en
